# Biologics for Psoriasis During the COVID-19 Pandemic

**DOI:** 10.3389/fmed.2021.759568

**Published:** 2021-12-06

**Authors:** Huanhuan Zeng, Siyu Wang, Ling Chen, Zhu Shen

**Affiliations:** ^1^School of Medicine, Zunyi Medical University, Zunyi, China; ^2^Department of Dermatology, Institute of Dermatology and Venereology, Sichuan Academy of Medical Sciences & Sichuan Provincial People's Hospital, Chengdu, China; ^3^Department of Dermatology, Daping Hospital, Army Medical University, Chongqing, China; ^4^School of Medicine, University of Electronic Science and Technology of China, Chengdu, China

**Keywords:** COVID-19, psoriasis, biologics, TNF, IL23, IL17, vaccination, SARS-CoV2

## Abstract

Coronavirus disease 2019 (COVID-19), a new form of acute infectious respiratory syndrome first reported in 2019, has rapidly spread worldwide and has been recognized as a pandemic by the WHO. It raised widespread concern about the treatment of psoriasis in this COVID-19 pandemic era, especially on the biologics use for patients with psoriasis. This review will summarize key information that is currently known about the relationship between psoriasis, biological treatments, and COVID-19, and vaccination-related issues. We also provide references for dermatologists and patients when they need to make clinical decisions. Currently, there is no consensus on whether biological agents increase the risk of coronavirus infection; however, current research shows that biological agents have no adverse effects on the prognosis of patients with COVID-19 with psoriasis. In short, it is not recommended to stop biological treatment in patients with psoriasis to prevent the infection risk, and for those patients who tested positive for SARS-CoV-2, the decision to pause biologic therapy should be considered on a case-by-case basis, and individual risk and benefit should be taken into account. Vaccine immunization against SARS-CoV-2 is strictly recommendable in patients with psoriasis without discontinuation of their biologics but evaluating the risk-benefit ratio of maintaining biologics before vaccination is mandatory at the moment.

## Introduction

The coronavirus disease 2019 (COVID-19) caused by severe acute respiratory syndrome coronavirus 2 (SARS-CoV-2) has spread across the globe rapidly since its outbreak (1, 2). Similar to severe acute respiratory syndrome coronavirus (SARS-CoV) and Middle East respiratory syndrome coronavirus (MERS-CoV), the SARS-CoV-2 can cause excessive and aberrant non-effective host immune responses that are associated with acute lung injury (ALI) and acute respiratory distress syndrome (ARDS) (3, 4). Ren et al. (5) identified five hyperinflammatory cell subtypes that might be the major sources driving the inflammatory storm in lung injury, including a subtype of macrophage (Macro_c2-CCL3L1), three subtypes of monocytes (Mono_c1-CD14-CCL3, Mono_c2-CD14-HLA-DPB1, and Mono_c3-CD14-VCAN), and neutrophils. These hyper-inflammatory subtypes highly express specific cytokines, for example, Macro_c2-CCL3L1, specifically expresses CCL8, CXCL10/11, and interleukin (IL)-6; Mono_c1-CD14-CCL3 uniquely expresses high levels of IL-1β, CCL20, CXCL2, CXCL3, CCL3, CCL4, HBEGF, and tumor necrosis factor (TNF); and neutrophils express cytokines including TNFSF13B, CXCL8, FTH1, and CXCL16. This is consistent with the research results of Blanco-Melo et al. (6).

Psoriasis is an immune-mediated inflammatory skin disease with erythema, papules, and scales as the main clinical manifestations, in which both genetic and environmental factors participate. A self-sustaining cycle of inflammation plays an important role in psoriasis pathogenesis, mediated mainly by T cells and cytokines such as TNF-α, IL-23, and IL-17 (7). With these cytokines as targets, biological agents have become a major innovation in the treatment of psoriasis in the past 20 years and drastically changed our ability to treat psoriasis and psoriatic arthritis. Until now, there are biologics in four different classes (anti-TNF-α, anti-IL-17, antiIL-12/IL-23p40, and anti-IL-23p19) have been approved for the treatment of moderate-to-severe psoriasis (8).

Biologics are considered to have high infection risks, and some studies found that the overall infection rate is higher than that of placebo (9, 10). However, it is inappropriate to speculate the susceptibility of SARS-CoV2 according to these previous studies because these studies did not analyze the risk of virus infection separately. More importantly, a published study suggested that the SARS, which has similar pathogenesis with SARS-CoV-2, may have a different immune response compared with other respiratory viruses (11). In the pandemic era, explaining the relationship between biologics and coronavirus infection is imperative. Our review will summarize key information that is currently known about the impact of biologics on the risk of SARS-CoV-2 infection and severe COVID-19 outcomes.

## Biological Agents for Psoriasis and COVID-19 Infection

### Anti-TNF-α

Tumor necrosis factor α (TNF-α) is an inflammatory cytokine produced by macrophages/monocytes during acute inflammation. It plays an important role in host defense against intracellular bacterial infections, such as *Mycobacterium tuberculosis* and *Listeria monocytogenes*, and it is indispensable in epithelial granuloma formation (12–15). Currently, four anti-TNF-α agents are in use for psoriasis: adalimumab, certolizumab pegol, etanercept, and infliximab (16). A fifth anti-TNF-α agent, golimumab, is currently approved for the treatment of psoriatic arthritis but not psoriasis (8).

The role of TNF-α in virus defense is complex, and different viruses seem to have different immune effects. An early *in vitro* study showed that H5N1 virus infection was capable of leading to highly excessive TNF-α secretion by macrophages, quantitatively similar to that seen after stimulation with lipopolysaccharide (17). This means that if TNF-α participates in the inflammatory cascade, which results in lung injury in virus infection, then TNF-α inhibition could have the potential to dramatically reduce this lung damage. This has been certificated in an animal trial, in which mice with lung disease caused by respiratory syncytial virus or influenza virus have a dramatic reduction of overall illness severity without interfering with viral clearance after anti-TNF antibody treatment (18). For SARS-CoV-2, higher serum levels of TNF-α have been observed in many patients with severe COVID-19 compared with individuals with mild disease (19, 20). Based on these findings, after the outbreak of the COVID-19, the use of TNF-α inhibition to treat this disease was proposed (21). However, the role of TNF-α in the inflammatory response is still unclear, and key questions are whether and when anti-TNF-α therapy should be given. Therefore, more research and clinical trials are needed to confirm the effectiveness of TNF-α blocking treatment in COVID-19.

The increased risk of opportunistic infections by anti-TNF-α therapies has been reported in patients with inflammatory bowel disease (22). However, there are no relevant research results on the risk of infection of SARS-CoV-2 for patients with psoriasis with anti-TNF-α therapy. Some current case reports show that patients with psoriasis receiving TNF-α inhibition therapy can recover from the infection, even without any clinical symptoms. Conti et al. (23) described a case series of four patients with psoriasis treated with biologics who had a risk contact with COVID-19. In the series, a 67-year-old woman receiving adalimumab since September 2019 was quarantined because of contact with three of her family members suffering from mild COVID-19. This patient with psoriasis did not develop any signs or symptoms of COVID-19 while continuing adalimumab therapy during her quarantine. Another case reported by Valenti et al. (24) presented a 57-year-old male patient with psoriasis and psoriatic arthritis treated with adalimumab since June 2018. He was confirmed with Sars-CoV-2 infection and hospitalized, and he soon recovered from his COVID-19. The resumed adalimumab treatment after discharge did not cause a relapse of COVID-19-related symptoms. It seems that anti-TNF-α use does not lead to a serious outcome for patients with COVID-19. However, ARDS has been reported in patients with psoriasis under anti-TNF-α therapy (etanercept), in which the patient was affected by multiple comorbidities including obesity, hypertension, diabetes, and chronic renal failure (25). The relation between anti-TNF-α and ARDS is still not clear. Investigations with higher evidence, such as cohort study, and systematic reviews are needed to clarify it.

### Anti-IL17A/IL17R

There are several anti-IL-17 agents approved for psoriasis treatment, including secukinumab, ixekizumab, and brodalumab. Both secukinumab and ixekizumab specifically target IL-17A, and brodalumab targets the IL-17 receptor A unit (IL-17RA), inhibiting IL-17A, IL-17F, and two other members of the IL-17 cytokine family (IL-17C and IL-17E or IL-25) (26). Bimekizumab, targeting both IL-17A and IL-17F, is in phase 3 clinical trial for psoriasis (27). The IL-17 family includes six IL-17-family ligands [IL-17A, IL-17B, IL-17C, IL-17D, IL-17E (IL-25), and IL-17F], and five receptors (IL-17RA, IL-17RB/IL-25R, IL-17RC, IL-17RD/SEF, and IL-17RE) (28). IL-17A (hereafter referred to as IL-17) is the most intensively studied, and it is produced by multiple immune cells including T cells, macrophages, dendritic cells (DCs), natural killer cells, natural killer T cells, lymphoid tissue inducer cells, and γδ-T cells (29).

IL-17 plays a vital role in protecting the host from infection, and this is particularly evident at the skin and mucosal sites, such as the lung, gut, and oral cavity. It performs immune defense functions mainly *via* stimulation of granulopoiesis and neutrophil trafficking and promotes the expression of various anti-microbial genes. However, IL-17 is not always beneficial in protecting the host from infection. In certain infectious settings, it can mediate pathogenic inflammatory responses and contribute to inflammatory injury secondary to infection (28). Its predominant role seems to be dependent on where the cytokine is expressed (the gut, lung, or skin) and what the precipitating trigger is. These two factors appear to influence whether the prevailing effect of its expression is protective or whether it leads to a detrimental hyper-inflammatory state (30).

Similar to TNF-α, the mean serum levels of IL-17 in the patients with COVID-19 were significantly higher than those observed in the control group. And systemic IL-17 level was observed to have a positive and significant correlation with TGF-β, which is seen as a predictive factor of disease severity in patients with COVID-19 (31–33). The synergistic effects with IL-6 to prevent apoptosis of infected cells and promote the virus persistence and stimulating downstream cytokine release may be a possible molecular mechanism in immune injury by virus (30, 34). These effects suggest that IL-17 may be related to cytokine storm and disease severity, and IL-17 inhibitors could be presented as promising targets for the prevention of aberrant inflammation and acute respiratory distress in COVID-19. Of note, there is a clinical trial on the safety and efficacy of ixekizumab treatment for patients with COVID-19 in progress in China (35).

Galluzzo et al. (36) conducted a 136-week, real-life study of 151 patients with moderate-to-severe plaque psoriasis being treated with secukinumab, and they found that there were no cases of confirmed infection with SARS-CoV-2 among 119 patients who continued to receive treatment with secukinumab. Only one patient had been placed in quarantine due to contact with a COVID-19 positive patient, and he completed the isolation period without infection. Balestri et al. (37) reported a patient with psoriasis infected with COVID-19 completely asymptomatic during ixekizumab induction treatment, and he recovered from COVID-19 without any antiviral therapy 1 month later. Mugheddu et al. (38) reported two patients with psoriasis infected with SARS-CoV-2 while on long-term secukinumab administration. They rapidly recovered from the infection between the two scheduled doses of secukinumab. For those who are elderly and affected by hypertension, which is both risk factors found to be associated, respectively, with overall case-fatality rate and severity of COVID-19, there seems still is a favorable outcome with secukinumab (39).

Current knowledge and clinical practice have shown that IL-17 inhibition will not interfere capacity of patients to develop excellent responses to SARS-CoV2. Therefore, it can be safely continued in patients with psoriasis exposed to COVID-19, with a favorable course and rapid recovery even in more critical patients.

There is no evidence that IL-17 inhibition can increase the risk of SARS-CoV-2 infection or lead to a severe outcome. However, Foti et al. (40) reported a contrary case in which a 57-year-old man with psoriatic arthritis who was treated with methotrexate and secukinumab reported COVID-19 symptoms and was tested for SARS-CoV-2 positive. This patient developed rapid worsening of clinical symptoms and resulted in ARDS. Unlike the previous findings, low IL-6 values were found at all stages of the disease in this patient, and the authors think other cytokines and mechanisms may have a role in this critical patient with COVID-19 who progressed to multiple organ dysfunction. In this case, the effect of methotrexate also should be taken into consideration. Methotrexate has been reported to significantly decrease IL-6 and TNF-α in T cells (41). This may lead to an insufficient immune response for virus defense. A meta-estimate on the risk of respiratory tract infections (RTIs) and symptoms in patients with psoriasis treated with IL-17 inhibitor biologics found an increased risk of RTIs compared with placebo (odds ratio, 1.56; 95% CI:1.04–2.33) (42). These findings indicate that it is necessary to evaluate the impact of IL-17 inhibitors on RTIs in the pandemic more meticulously. And clinicians should use their clinical judgment to help patients make clinical decisions about whether to discontinue biological agents.

### Anti-IL23

IL-23 is a heterodimer composed of a p40 subunit also found in IL-12 and a p19 subunit exclusive to IL-23 (43). IL-23 is involved in promoting chronic tissue inflammation during infection, granuloma formation, and autoimmunity by maintaining the amplification of Th17 and cytotoxic T-cell type 17 (Tc17) responses. The IL-23/Th17 immune axis has been identified as a major immune pathway in psoriasis pathogenesis, in which IL-23 plays a predominant driver (44). There are currently four agents that target IL-23 in clinical use for psoriasis: ustekinumab, which blocks the common p40 subunit of IL-12 and IL-23, and guselkumab, risankizumab, and tildrakizumab, which target the p19 subunit of IL-23. A fourth anti-IL-23p19 biologic, mirikizumab, is currently in phase 3 clinical studies (8).

Different from TNF-α and IL-17, which respond to coronavirus and viral pneumonia, IL-23 does not seem to contribute to these complications, neither to have a major impact on anti-viral immunity (23). The safety of IL-23 inhibitors during the COVID-19 epidemic has also been reported. A multicenter study conducted during the first 4 months of the pandemic in Central Italy showed excellent tolerance and safety of risankizumab. In the study, only one patient (1.8%) experienced upper RTI, three patients (5.3%) had contact with SARS-CoV-2-infected subjects, and no one experienced SARS-CoV-2 infection among 57 patients (45). These results indicated that the use of IL-23 inhibitors will not increase the rate of SARS-CoV-2 infection. A series of clinical case reports also indicate that IL-23 inhibitors will not allow patients to experience a more serious disease process or outcome. Patients who suffered COVID-19 during their anti-IL-23 treatment achieved full recovery from COVID-19 and remained asymptomatic or developed mild symptoms, even some at risk of severe COVID-19 development (46–48). As a driver for IL-23/Th17 immune axis, IL-23 plays a role by increasing IL-17 in psoriasis pathogenesis. Theoretically, it has little impact on interferon-γ or mucosal immune, which is important for virus defense. So, this may contribute to the low SARS-CoV-2 infection risk, and its attenuation effects on IL-17 may result in a milder manifestation of COVID-19.

## The Relationship Between Psoriasis and COVID-19

### The Risk of COVID-19 Infection and Outcome in Psoriasis on Biologics Therapy

As early as when the COVID-19 epidemic broke out, research on the safety of biological agents during the special period began to appear. The results of an observational study of 107 patients with psoriasis treated with biologics conducted in Wuhan showed that none of the 107 patients with psoriasis were diagnosed with COVID-19, including 55 (51.4%) patients who were either residents or had traveled to Wuhan after November 2019. Four patients (3.7%) had a history of close contact with patients infected with COVID-19, but none of these patients developed any COVID-19 symptoms (49).

As the epidemic spreads globally, more reports have emerged describing the susceptibility to COVID-19 and the severe clinical course of the disease. The results of several cohort studies from Italy conducted by Gisondi et al. (50) show that, compared with the general population, the use of biological agents for patients with psoriasis does not increase the infection rate, hospitalization rate, and mortality of COVID-19. They found the COVID-19 incidence rate (IR) was 9.7 (95% CI 3.9–20.1) per 10,000 person-months in a 1,830-patient cohort and 11.5 (95% CI 11.4–11.7) per 10,000 person-months in the general regional population. The IR of hospitalization for COVID-19-related pneumonia and COVID-19-related death was 6.5 (95% CI 2.0–15.6) and 0 (95% CI 0–10.4) per 10,000 person-months in their cohort, lower than the general population with 9.6 (95% CI 9.4–9.7) and 1.16 (95% CI 1.10–1.21) per 10,000 person-months. Here, we speculate that there may be two factors contributing to these results. First, long-term use of anti-inflammatory agents (including biologics) for patients with psoriasis may reduce the release of inflammatory cytokines and alleviate the inflammatory damage; moreover, patients with psoriasis may tend to have stricter personal protective measures and social isolation for fear of infection. Similar observations are also shown in their other two papers (51, 52). Another observational study from Italy also observed that the incidence of COVID-19 observed in the cohort of patients with psoriasis (0.2%) is similar to that seen in the general population (0.31%), and the course of the disease was mild in most patients (53) and similar observational conclusions have been confirmed in other studies (54–57).

When compared with patients with psoriasis without biological agents, there comes to a consistent conclusion. Mahil et al. (58) analyzed the factors for adverse outcomes in 374 patients with psoriasis infected with COVID-19, and they found biologic use was associated with a lower risk of COVID-19-related hospitalization than with the use of non-biologic systemic therapies. A multicenter study in Istanbul recorded demographics and disease characteristics of 1,322 patients with psoriasis with a semi-structured questionnaire. The results of the study showed that 23 patients have been diagnosed or suspected of COVID-19, and the rate of distribution of biological treatment in COVID-19(–) and COVID (+) groups showed no statistically significant difference. Hospitalization from COVID-19 between patients using biologics (*n* = 9) and those not using them (*n* = 14) also did not have a statistically significant difference. These data further indicate that biologics do not have any adverse impact on COVID-19 infection or outcome in patients with psoriasis. The current research results seem to be encouraging; however, clinicians should be cautious when giving treatment recommendations based on this because these studies have some limitations on the whole, such as lack of standardization for the control group, insufficient sample size, and confounding factors that are no get controlled. Therefore, rigorously designed randomized controlled trials with larger samples are needed to further confirm these conclusions.

### The Impact of Psoriasis Itself on COVID-19 Infection

Most of the current research focuses on the impact of psoriasis treatment or comorbidities on the COVID-19, and there are few studies on the impact of psoriasis itself on the disease. Research shows psoriasis is one of the most common dermatological diseases in patients with COVID-19 who have had dermatological diseases for the last 3 years. Tan et al. (59) also had a similar finding. They studied 133,589 patients diagnosed and 48,418 patients hospitalized with COVID-19 with prevalent autoimmune diseases, and they found that the most prevalent autoimmune conditions among patients with COVID-19 were psoriasis (3.5–32.5%), rheumatoid arthritis (3.9–18.9%), and vasculitis (3.3–17.6%). These can pose a possibility that patients with psoriasis may be more vulnerable to the COVID-19. But the difference in the morbidity of prevalent autoimmune diseases should be taken into account when explaining the data. However, some subsequent studies showed different results. Yiu et al. (60) performed a cross-sectional study to investigate the risk of COVID-19 infection in psoriasis. They found among 1,427 patients with psoriasis, there were only 12 patients diagnosed with COVID-19, and no statistically significant elevated risk for infection with COVID-19 was found (unadjusted odds ratio, OR 0.60 [95% CI 0.33, 1.08], complete case adjusted OR 0.98 [95%CI 0.46, 2.08], and MI adjusted OR 0.50 [95% CI 0.28, 0.92]). A retrospective cohort study conducted by Raiker et al. (61) suggested that patients with Pso-COVID and PsoA-COVID were not at higher risk for severe COVID complications. The history of immunosuppressant use in both cohorts also revealed no higher risk in COVID complications. Compared with patients with non-Pso-COVID, patients with Pso-COVID had a similar risk of hospitalization (0.90 [0.78–1.03]), sepsis (0.78 [0.54–1.14]), mortality (0.82 [0.57–1.19]), and severe COVID (0.77 [0.58–1.03]), even had statistically significant lower risk of ARDS (0.51 [0.30–0.90]), and mechanical ventilation (0.65 [0.45–0.95]). As currently available evidence is relatively scarce and has certain limitations, further research in larger cohorts with representative denominators is needed to confirm this finding and to observe the longer-term impacts.

### The Impact of COVID-19 Infection on Psoriasis

The host cell entry of SARS-CoV-2 depends on the angiotensin-converting enzyme 2 (ACE2) and transmembrane protease serine 2 (TMPRSS2). ARS-CoV-2 enters the host cell by its spike protein interacting with the receptor ACE2 present on the host cell surface. TMPRSS2 plays a vital role in cleaving the SARS-CoV-2 spike protein, thereby enabling the virus to enter the host cell by endocytosis (62, 63). Since the outbreak of COVID-19, numbers of case reports and clinical series have described a complex spectrum of skin manifestations associated with the infection (64). Sun et al. (65) found that the co-expression of ACE2 and TMPRSS2 was particularly found in the granulosum of skin, so they proposed the hypothesis that skin is a potential host of SARS-CoV-2 and there is a potential risk of SARS-CoV-2 transmission *via* wounded skin in those with skin manifestations of the disease. Controlled studies on patients with psoriasis have shown a significantly increased expression of ACE2 (*p* = 0.009) in lesion skin compared with healthy controlled skin, but no significant difference was observed for TMPRSS2 (*p* = 0.19) (63, 66). These findings suggest that whether the skin lesions of patients with psoriasis are the target of SARS-CoV-2 infection still needs further investigation.

Psoriasis is a chronic inflammatory disease that can be aggravated by drug, stress, and viral infection, especially rhinovirus and coronavirus (67–70). In the era of the pandemic, Kutlu and Metin et al. (71) presented for the first time a case of psoriasis potentially triggered by COVID-19 infection and hydroxychloroquine. They reported a 71-year-old woman who had a history of psoriasis but without skin lesions when admitted to the pandemic clinic with the diagnosis of COVID-19. The patient had a recurrence of psoriasis on the 4th day of COVID-19 treatment with oseltamivir and hydroxychloroquine. Similarly, the exacerbation of pustular psoriasis and psoriatic arthritis also could be observed in COVID-19 who were treated with hydroxychloroquine (72–74). These suggested that the exacerbation of psoriasis was due to the use of hydroxychloroquine, but do not rule out the possibility that the COVID-19 virus might play a role in the process. Subsequent case reports provided some evidence for the vision. A 38-year-old man who confirmed COVID-19 infection was presented had an acute guttate flare of chronic psoriasis during his quarantine without any treatment (75). Zhou et al. (76) conducted an observational study on 18 patients with psoriatic arthritis and found an increased disease activity in psoriatic arthritis (DAPSA) score and statistically significant increases of swollen and tender joint count following COVID-19 infection.

A possible mechanism for psoriatic flares following COVID-19 infections is the induction of a hyperinflammatory state. It has been shown that binding of the coronavirus spike protein to the ACE2 receptor would result in ACE2 downregulation and then lead to excessive production of ACE. So, some researchers speculate that COVID-19 infection may aggravate the psoriatic condition and accompany a higher incidence of cardiovascular events in psoriasis as ACE has been proposed in the process of inflammation (77).

Based on these findings, it is important to pay attention to psoriasis when patients with COVID-19 receive treatment with hydroxychloroquine, and it is recommended to discontinue the use of hydroxychloroquine in patients with COVID-19 who develop psoriasis or experience a recurrence of psoriatic skin lesions (72). In addition, fish oil supplementation can be considered in the treatment regimen of psoriasis subjects in case of COVID-19 infection, as it can inhibit ACE activity and decrease symptoms in psoriasis subjects (78).

## Recommendations for the use of Biologics During the Pandemic of COVID-19

According to the recommendations of major global dermatological associations, patients who had not reported clinical symptoms or close contact with a confirmed or probable COVID-19 case in the last 14 days can continue biologic therapy. It is advisable to discontinue or postpone biological treatment in patients with confirmed SARS-CoV-2 infections until COVID-19 is fully cured. For those patients who are qualified for biological treatment but have not yet started, it is advisable to carefully assess the balance of benefits and risks of treatment for each patient. In populations with a high risk for severe COVID-19, a postponement of biological treatment or other therapeutic options should be considered (79–81). The National Psoriasis Foundation COVID-19 Task Force has reiterated a similar point in the guidance for the management of psoriatic disease during the pandemic. It is recommended that patients who are not infected with SARS-CoV-2 continue their biologics for psoriasis in most cases. Shared decision-making between clinician and patient is recommended to guide discussions about the use of systemic therapies during the pandemic (82).

## SARS-CoV-2 Vaccination in Patients With Psoriasis Under Biologic Therapy

Since the outset of the COVID-19 pandemic, COVID-19 vaccines were being developed around the world. The COVID-19 vaccines currently allowed for emergency use worldwide are mainly mRNA vaccines, adenovirus vector vaccines, and whole-virion inactivated vaccines (83).

Clinical trials showed a high efficacy rate of these vaccines in protection against COVID-19 and no major safety concerns (84–88). However, there are currently no data on the efficacy and safety of COVID-19 vaccines in patients with psoriasis treated with biologic drugs as patients on immunosuppressive therapy were excluded from clinical trials. Some major international scientific societies, for example, National Psoriasis Foundation, recommend the use of the SARS-CoV-2 vaccine even in patients undergoing biological therapy without the necessity to discontinue the therapy (89, 90). Some patients with psoriasis are still reluctant to get vaccinated out of concern about its safety and efficacy, as there have been reported that patients with psoriasis may have flare-ups after vaccination, and concomitant immunosuppression may impair the immune response to vaccination (91–93).

Damiani et al. (94) and Pacifico et al. (95) have preliminarily confirmed the safety and efficacy of the vaccines in their case series report. Patients with psoriasis under biologics and apremilast underwent Pfizer mRNABNT162b2 and AZD1222 (AstraZeneca-Oxford vaccine), and they did not experience any psoriasis flare or cutaneous manifestations. All patients developed IgG anti-S1-Receptor Binding Domain (RBD) of SARS-CoV-2 without discontinuation or modification of their therapy. A survey on the antibody responses to single-dose mRNA vaccines in patients receiving immunomodulatory drugs suggested that 15% of patients failed to detect antibody response to single-dose BNT162b2 or AZD1222 vaccines; 41% had no detectable anti-S1 IgG. Compared to biologics, non-biologic immunomodulators, such as methotrexate, had a lower level of antibody response. This contrasts with data from healthy populations, which show close to 100% (96), and then Geisen et al. (97) evaluated antibody responses following the second dose of mRNA vaccines in a cohort study of 42 controls and 26 patients with immunomodulatory drugs. The result showed that anti-SARS-CoV-2 antibodies could be detected in all participants. But lower anti-S IgG levels also could be detected in patients receiving immunomodulators. Side effects were comparable in both groups. No severe adverse effects were observed, and no patients experienced a disease flare.

These show that immunosuppressed patients may have an impaired immune response to SARS-CoV-2 mRNA vaccines, but the safety is proven. When it comes to a specific biological agent, the current evidence is limited. The impact of anti-TNF-α agents on vaccine response is controversial (98, 99). The meta-analysis performed by Subesinghe et al. (100) showed that anti-TNF-α therapy did not impair influenza vaccine responses. For anti-IL17 agents, the current literature shows that they do not seem to affect the humoral immune response to non-live vaccines. In a randomized, open-label, parallel-group study Gomez et al. (101) found, compared with the control group, the subjects who received 160 mg ixekizumab subcutaneously 2 weeks before vaccination and 80 mg ixekizumab on the day of vaccination had a comparable level of immune response to the tetanus vaccine and the Streptococcus pneumoniae vaccine. Similarly, a cohort study aimed to compare the basal antibody titers against the three influenza vaccines between psoriatic arthritis and ankylosing spondylitis who were receiving treatment with secukinumab and healthy volunteers were included. This research has reached a consistent conclusion that secukinumab did not influence the response to the influenza vaccine [relative risk, RR: 1.09 (95% CI 0.58–2.07) for h1N1, RR: 1.53 (95% CI 0.15–15.0) for h3N2, and RR: 0.72 (95% CI 0.32–1.83 for B strain)] (102).

## Conclusion and Discussion

With the COVID-19 spreading worldwide rapidly, the biological treatment of psoriasis has become a topic of great concern. At present, there is a lack of evidence for one or a class of biological agents on the impact of SARS-CoV-2 infection. Most of the existing evidence is based on clinical case reports. Therefore, it is difficult to evaluate which biological agent has better safety for COVID-19. However, from the current unclassified research, the biological treatment of psoriasis does not seem to have a significant impact on the COVID-19. Regardless of whether biological agents have been used, patients with psoriasis were not at higher risk for severe COVID complications. However, COVID-19 infection and use of hydroxychloroquine seem to be related to the recurrence or exacerbation of psoriasis, in addition, patients with psoriasis may be at a higher incidence of cardiovascular events in case of COVID-19 infection.

This emphasizes the importance of patients with psoriasis to prevent SARS-CoV-2 infections. Vaccination still is an effective measure to prevent the spread of infection, and patients with psoriasis are advised to be vaccinated without discontinuing their biological treatment. If the situation permits, it is best to vaccinate before starting biological treatment, because the current evidence suggests that the use of immunosuppressive agents may reduce the vaccine immune response to a certain extent. Related dermatological associations and clinical guidelines also recommend that undiagnosed patients should continue their biological therapy. For high-risk patients (older age, with comorbidities, or metabolic disorders such as diabetes and obesity), discontinue decision should be made on the evaluation of the balance of benefits and risks, and the risk of disease relapse and retreatment failure also should be taken into consideration. There are few relevant studies on psoriasis during the pandemic, and the current evidence has certain limitations. Therefore, it is necessary to be cautious when making clinical decisions. More prospective studies with higher levels of evidence are needed to support clinical decision-making.

## Author Contributions

ZS, HZ, SW, and LC contributed to conception and design of the study. HZ drafted the manuscript. SW, LC, and ZS contributed to the critical revision of the manuscript. HZ, SW, LC, and ZS contributed to the literature retrieval. All authors approved the submitted version.

## Funding

This work was supported by National Natural Science Foundation of China (Nos. 81771783 and 82073444).

## Conflict of Interest

The authors declare that the research was conducted in the absence of any commercial or financial relationships that could be construed as a potential conflict of interest.

## Publisher's Note

All claims expressed in this article are solely those of the authors and do not necessarily represent those of their affiliated organizations, or those of the publisher, the editors and the reviewers. Any product that may be evaluated in this article, or claim that may be made by its manufacturer, is not guaranteed or endorsed by the publisher.
